# Supplementary material for: Molecular variation in a functionally divergent homolog of FCA regulates flowering time in Arabidopsis thaliana
Source: Nat Commun. 2020 Nov 17;11:5830. doi: 10.1038/s41467-020-19666-0 (PMC7673134; doi:10.1038/s41467-020-19666-0)
Supplement: Supplementary file 1 — Supplementary Information [file 41467_2020_19666_MOESM1_ESM.pdf]

Molecular variation in a functionally divergent homolog of FCA regulates flowering time in *Arabidopsis thaliana*

Wang and Tao *et al.*

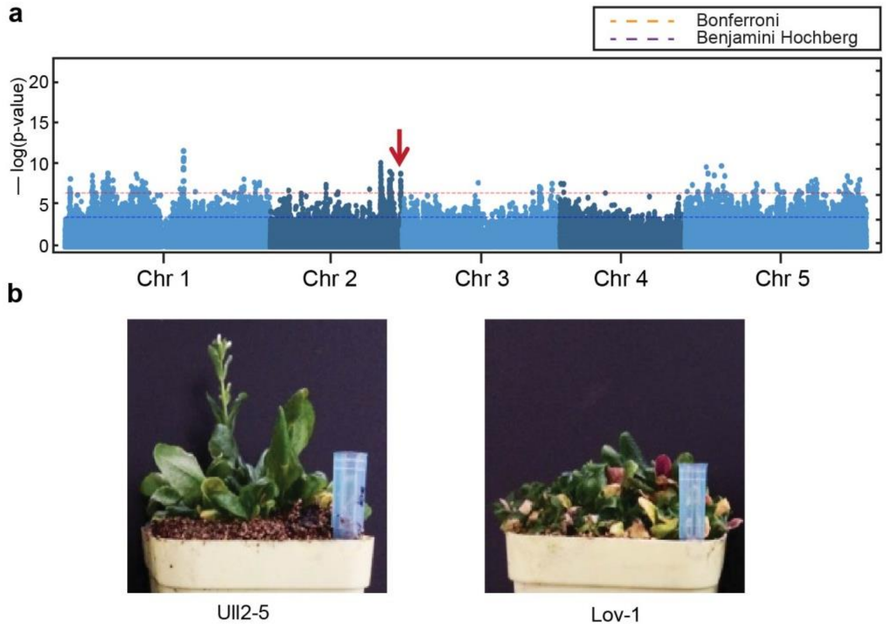

Supplementary Fig. 1. GWAS analysis of *FLC* expression without population structure correction and flowering time of Ull2-5 and Lov-1 accessions. a GWAS without population structure correction of *FLC* expression (T30/NV). The arrow indicates the At2g47310 genomic region. **b** The Lov-1 accession flowered later than Ull2-5 after 4 weeks of vernalization.

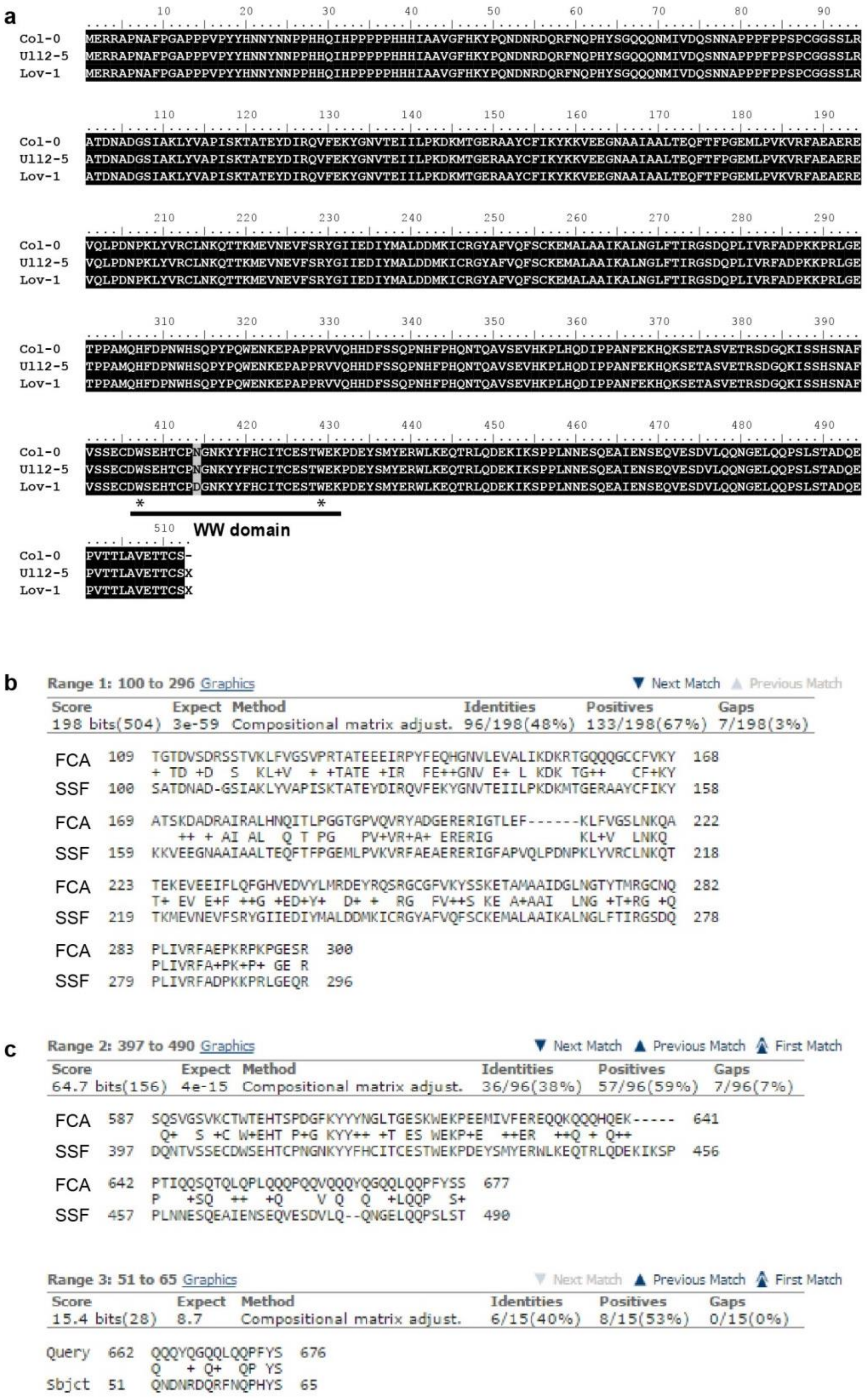

Supplementary Fig. 2. The amino acid variant N414D in the WW domain of SSF proteins. a The amino acid sequences of the SSF proteins of Col-0, Ull2-5, and Lov-1 accessions were aligned. The N414D and conserved tryptophans in the WW domain are highlighted. **b**, **c** Alignment of SSF with the FCA protein sequence. Only the two conserved RRM (**b**) and WW (**c**) domains are shown.

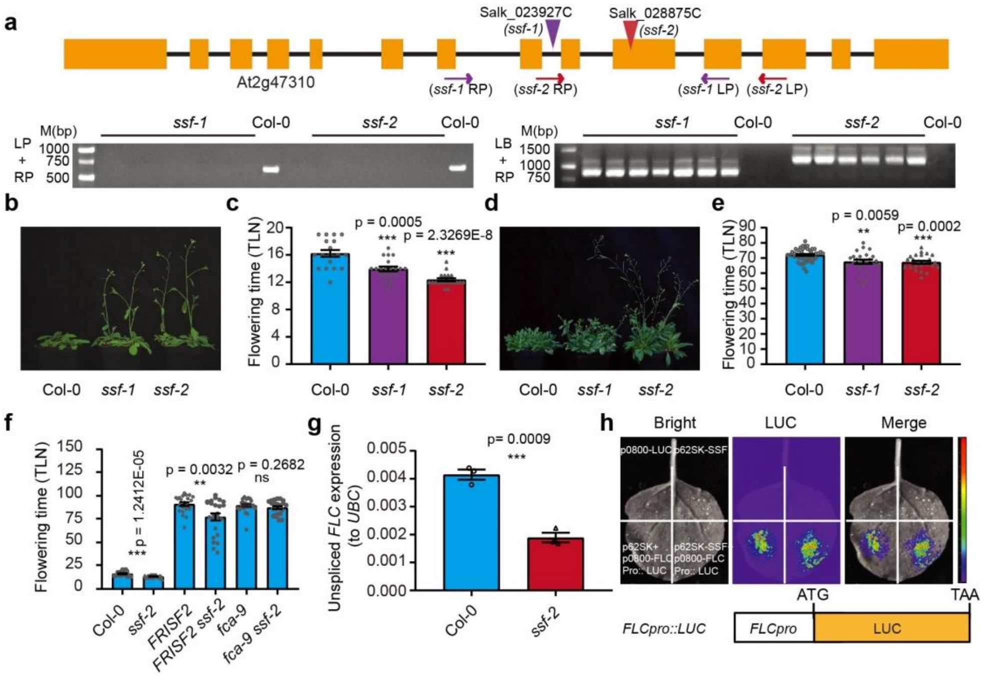

Supplementary Fig. 3. Characterization of the function of SSF. a Gene structure of *SSF* and genotyping confirmation of the *ssf* mutants. Col-0 is shown as a negative control. The primer positions are shown in the figure. LB indicates the primer anchored on the T-DNA. **b**-**e** Both *ssf-1* and *ssf-2* flowered earlier than Col-0 under long-day (**b**, **c**) and short-day (**d**, **e**) growth conditions (n=20). **f** Flowering time comparison between *ssf-2* and related double mutants (n=24). **g** The expression level of unspliced *FLC* was also lower in *ssf-2* mutant than in wild-type Col-0 (n=3). The *UBC* gene was used as an internal control. **h** SSF promotes *LUC* expression driven by the *FLC* promoter. The combination of *Agrobacterium* for cotransformation in *N. benthamiana* leaves is illustrated on the bright-field image on the left side. These experiments were repeated at least three times, and consistent results were obtained. In (**c**, **e**, **f**, **g**), data are presented as mean ± SEM. Asterisks indicate significant differences (** p<0.01, *** p<0.001, ns, not significant; two-tailed unpaired t-test). Source data are provided as a Source Data file.

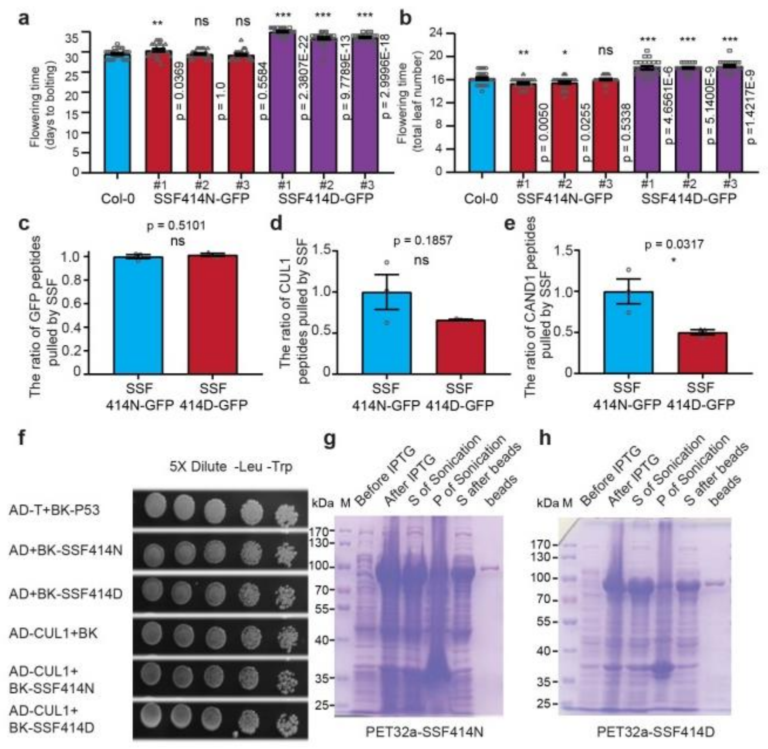

Supplementary Fig. 4. Pulldown and Y2H analysis of SSF-414N and SSF-414D with CUL1 and purification of the SSF protein in *E. coli*. a, b SSF-GFP constructs rescued the *ssf-2* flowering time phenotype, and SSF414D-GFP caused later flowering than SSF414N-GFP, as shown by the days to bolting (**a**) and total leaf number (**b**) (n=24). **c-e** The proportion of proteins pulled down by SSF (n=3). The peptide number of GFP (**c**), CUL1 (**d**), and CAND1 (**e**) was normalized to that of SSF from the pulldown complex. GFP is shown as an internal control. The raw data for the protein pulldown are included in Supplementary Table 6. **f** The control for the Y2H analysis to examine the SSF and CUL1 interaction shown in Fig. 4a. Five-fold dilutions of yeast cells were placed on SD medium lacking Trp and Leu. The interaction of T and P53 served as a positive control. The interactions between the empty vector AD with SSF414N or SSF414D and the empty vector BK with AD-CUL1 were used as negative controls. **g**, **h** SSF414N-His and SSF414D-His proteins purified using a prokaryotic (*E. coli*) system. The lanes from left to right are as follows: before IPTG induction, after IPTG induction, supernatant from sonication, pellet from sonication, supernatant after bead purification, bead-purified protein. These experiments were repeated at least three times, and consistent results were obtained. In (**a-e**), data are presented as mean ± SEM. Asterisks indicate significant differences (* p<0.05, ** p<0.01, *** p<0.001, ns, not significant; two-tailed unpaired *t*-test). Source data are provided as a Source Data file.

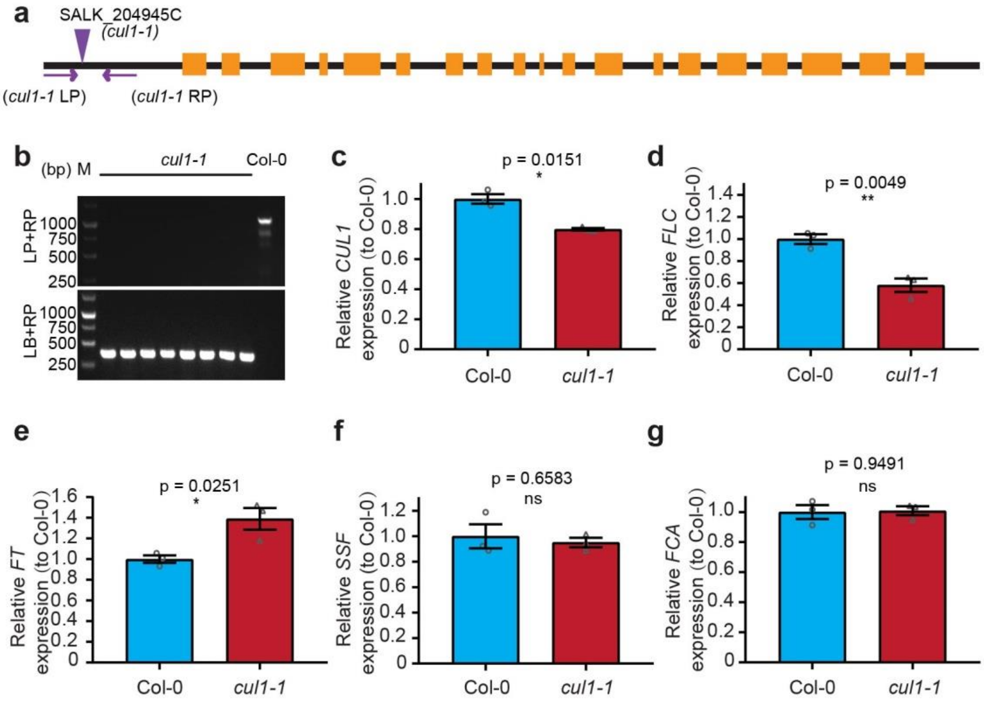

Supplementary Fig. 5. CUL1 regulates flowering time by affecting *FLC* and *FT* expression. a The gene structure and position of the genotyping primers are shown. Yellow boxes represent exon 2, and horizontal lines represent introns. **b** Identification of *cul1-1* mutants by PCR. Col-0 was used as a control. **c** *CUL1* expression was downregulated in the *cul1-1* mutant. **d** *FLC* expression was downregulated in the *cul1-1* mutant. **e** *FT* expression was upregulated in the *cul1-1* mutant. **f**, **g** *SSF* (**f**) and *FCA* (**g**) expression was not influenced by *CUL1* mutation. *UBC* was used as an internal control. These experiments were repeated at least three times, and consistent results were obtained. In (**c-g**), data are presented as mean ± SEM (n=3 biological replicates). Asterisks indicate significant differences (* p<0.05, ** p<0.01, ns, not significant; two-tailed unpaired t-test). Source data underlying Supplementary Figure 5b-g are provided as a Source Data file.

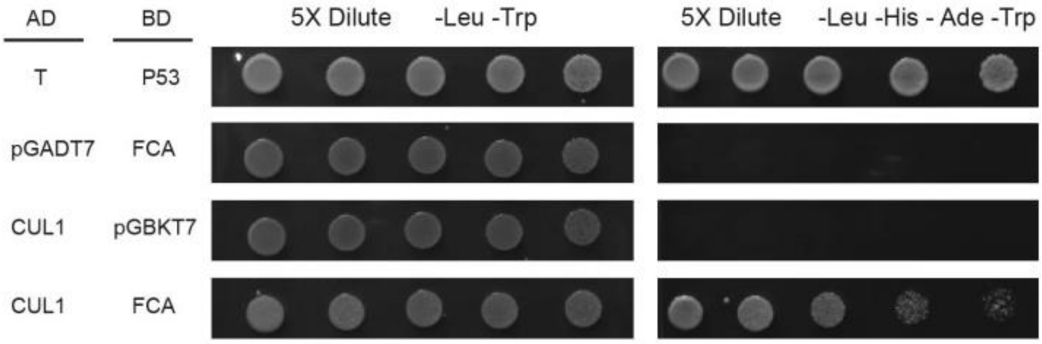

Supplementary Fig. 6. CUL1 interacts with FCA in yeast. The CUL1 and FCA interaction was examined by a Y2H assay. Five-fold dilutions of yeast cells were placed on SD medium lacking Trp and Leu and SD medium lacking Trp, Leu, His, and Ade. The interaction of T and P53 was used as a positive control. The interactions between the empty vector AD with FCA and the empty vector BK with AD-CUL1 were negative controls. Source data are provided as a Source Data file.

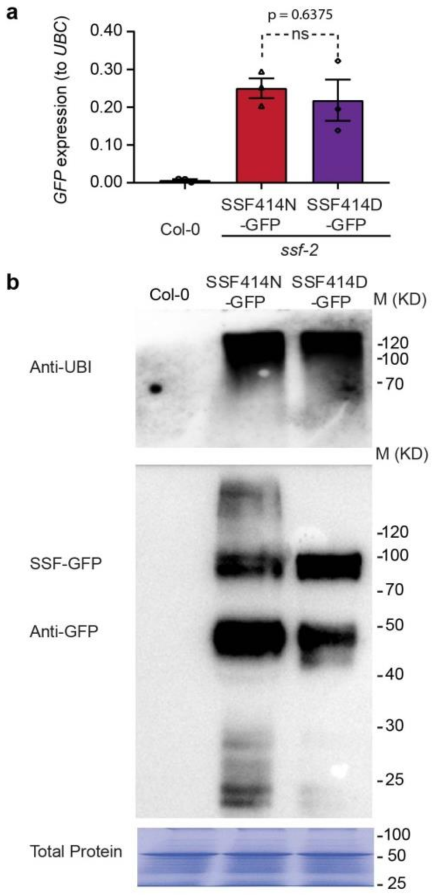

Supplementary Fig. 7. Gene expression and protein analysis in SSF414N-GFP and SSF414D-GFP transgenic plants. a *GFP* expression analysis in SSF414N-GFP and SSF414D-GFP complementation plants. Total RNA was extracted from seedlings after two weeks of growth under long-day growth conditions. The *UBC* gene was used as an internal control for RT-qPCR assays. Data are presented as mean ± SEM (n=3 biological replicates). (ns, not significant; two-tailed unpaired *t*-test). **b** In vivo detection of SSF-GFP in Col-0, SSF414N-GFP, and SSF414D-GFP plants. Total protein was extracted from whole seedlings of 14-day-old Col-0, SSF414N-GFP, and SSF414D-GFP transgenic seedlings and then incubated with GFP-Trap (ChromoTek, gtma-20) to enrich GFP-fused SSF. SSF-GFP was detected with an anti-GFP antibody (Roche, 11814460001). The polyubiquitination of SSF was detected with an anti-ubiquitin antibody [Ubi-1] (Abcam, ab7254). Total protein was used as a loading control. These experiments were repeated at least three times, and consistent results were obtained. Source data are provided as a Source Data file.

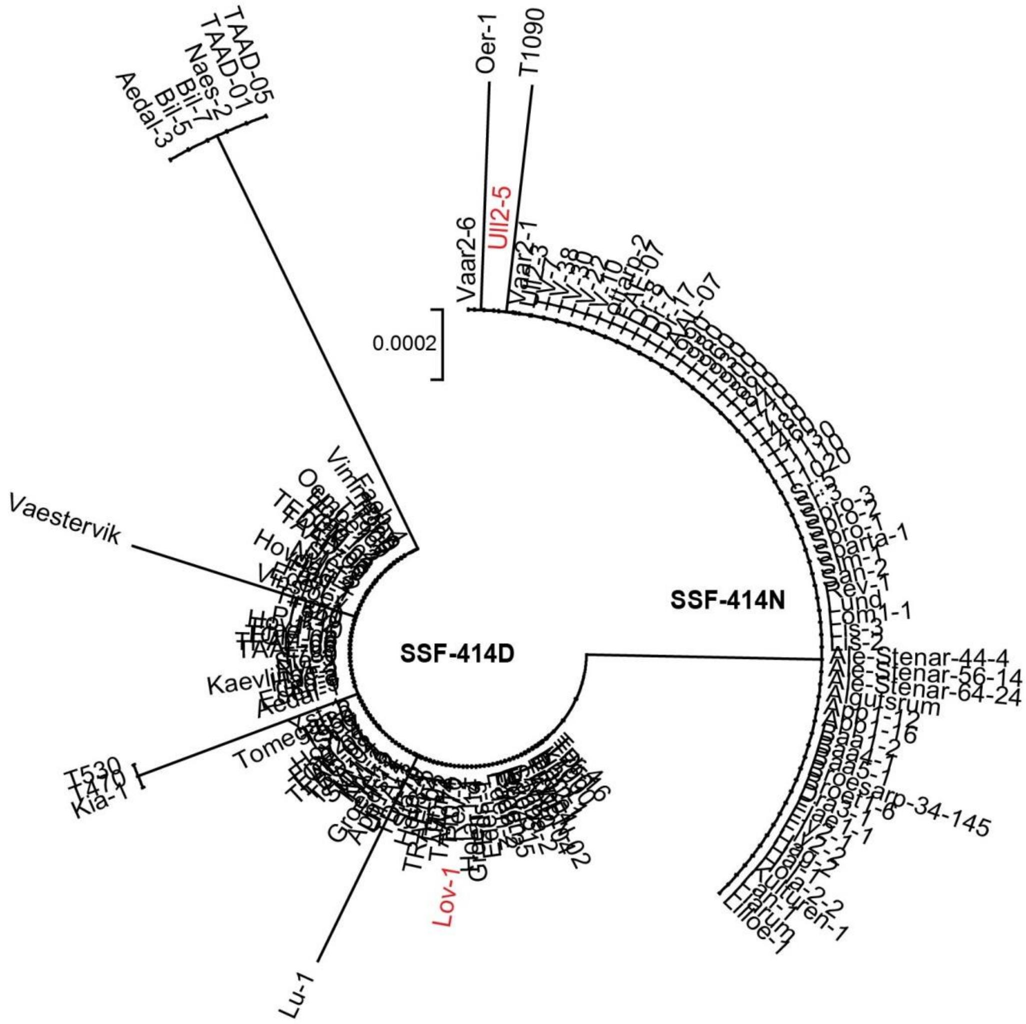

Supplementary Fig. 8. A neighbor-joining phylogenetic tree of the protein sequences from 169 Swedish *Arabidopsis* accessions. The representative accessions Ull2-5 and Lov-1 are shown in red. Accessions differing in the SSF polymorphism, SSF-N414D, grouped into two major haplotypes. The scale bar illustrates the difference between two sequences.

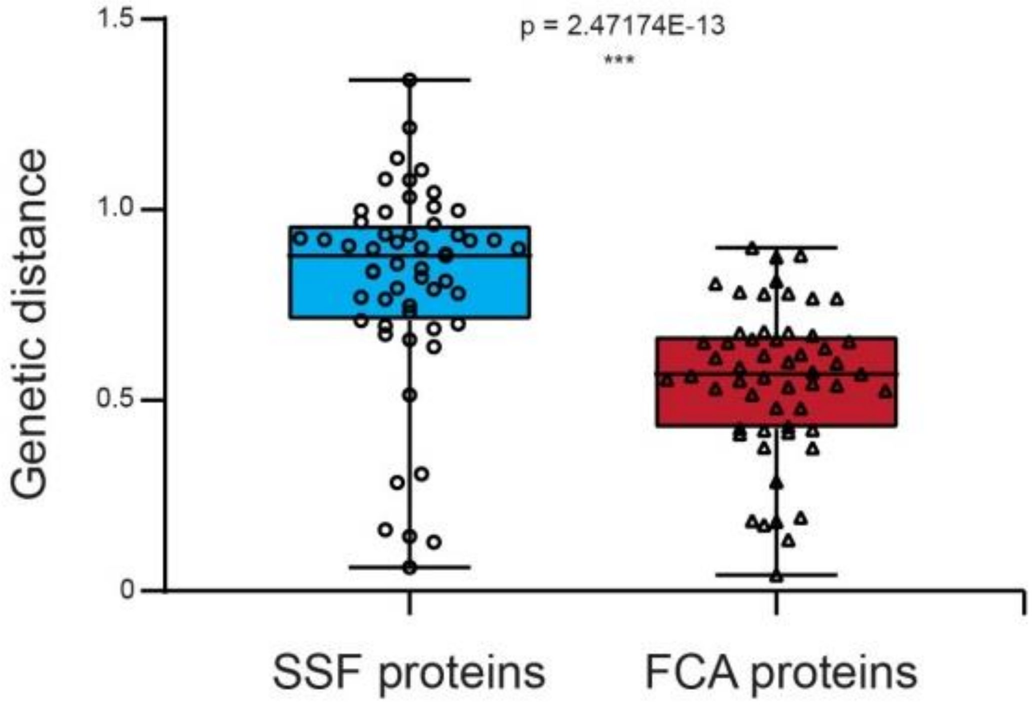

Supplementary Fig. 9. Comparison of the genetic distance between the FCA and SSF proteins. n=55. Box plot: lower vertical bar = sample minimum, lower box = lower quartile, middle line = median, upper box = upper quartile, upper vertical bar = sample maximum, single points = outliers. Asterisks indicate a significant difference (*** $p<0.001$; two-tailed unpaired *t*-test). Source data are provided as a Source Data file.

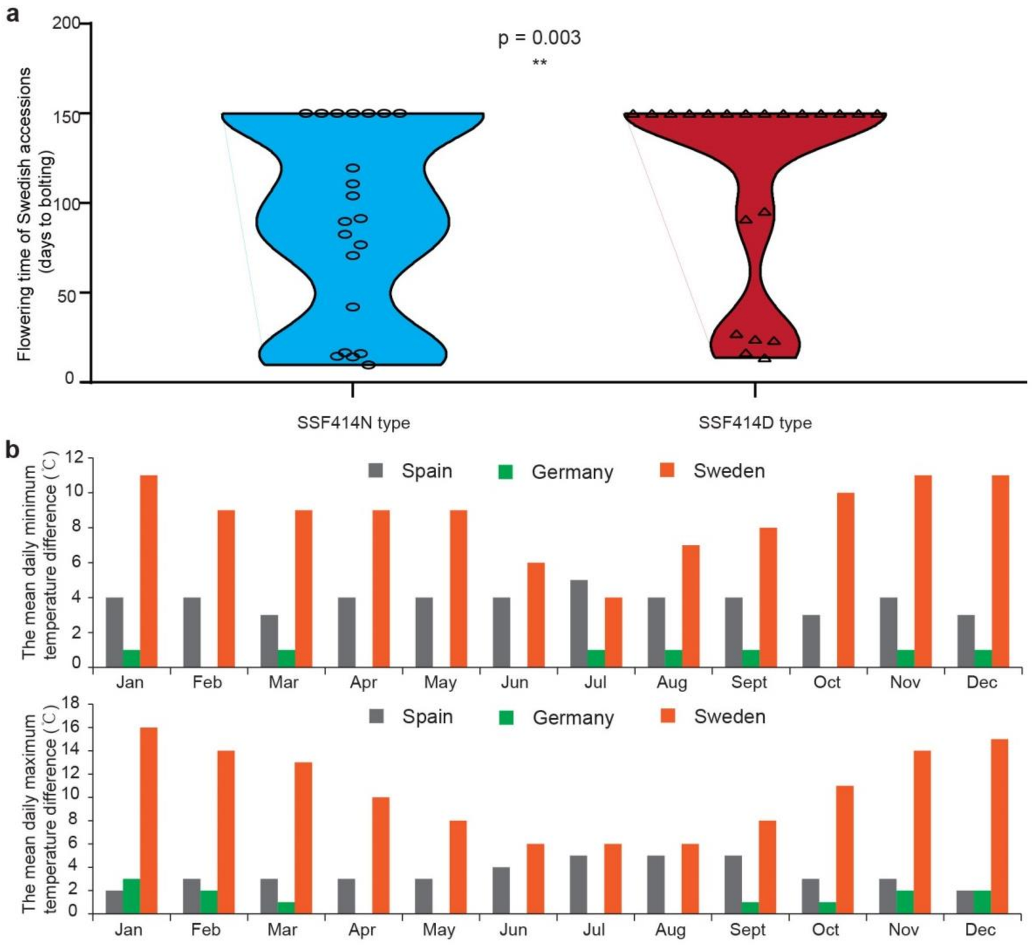

Supplementary Fig. 10. Flowering time of *Arabidopsis* accessions and growth environment comparison among Sweden, Spain, and Germany. a The Swedish accessions with SSF414N flowered significantly earlier than those with SSF414D (n of SSF414N=21, n of SSF414D=22). Asterisks indicate significant difference (** p<0.01; one-tailed unpaired moss extreme significance test). Note: The flowering time of 150 days means that the plant had not flowered when the experiment was terminated. **b** N. Sweden has a distinct ambient temperature compared with S. Sweden, whereas in Spain and Germany, the temperature difference between northern and southern areas is small. Source data are provided as a Source Data file.

Supplementary Table 1. List of the flowering time-associated genes in the GWAS and QTL analysis.

Name	AGI number	Chr.	Position start	Position end	Annotation
HOS1	At2g39810	2	16619910	16625135	HIGH EXPRESSION OF OSMOTICALLY RESPONSIVE GENES 1
ELF4	At2g40080	2	16741372	16741990	EARLY FLOWERING 4
SHP2	At2g42830	2	17827442	17831090	SHATTERPROOF 2
PIF4	At2g43010	2	17893504	17896127	PHYTOCHROME INTERACTING FACTOR 4
FPA	At2g43410	2	18033474	18038066	FPA
HUB1	At2g44950	2	18542213	18548591	HISTONE MONO-UBIQUITINATION 1
AGL6	At2g45650	2	18811424	18813596	AGAMOUS-LIKE 6
SOC1	At2g45660	2	18814612	18818094	SUPPRESSOR OF OVEREXPRESSION OF CO 1
CCA1	At2g46830	2	19252753	19255978	CIRCADIAN CLOCK ASSOCIATED 1
SSF	At2g47310	2	19430754	19434347	SISTER OF FCA (in this study)

Supplementary Table 2. Raw data for *FLC* expression in the F2 population.

	lines	*UBC*		*FLC*		Average cp		Δcp	*FLC* expression
		cp	cp	cp	cp				
**F2-Ull2-5 type**	F2#18	23.88	24.8	18.9	18.86	24.34	18.88	-5.46	44.02
	F2#36	25.56	25.68	19.9	19.99	25.62	19.95	-5.68	51.09
	F2#62	25.12	25.5	19.75	19.82	25.31	19.79	-5.53	46.05
	F2#115	25.23	25.47	19.27	19.29	25.35	19.28	-6.07	67.18
	F2#124	24.07	25.01	18.75	18.98	24.54	18.87	-5.68	51.09
	F2#138	25.1	25.21	20.11	20.15	25.16	20.13	-5.03	32.56
	F2#153	24.75	24.9	18.95	19	24.83	18.98	-5.85	57.68
	F2#158	24.59	24.59	19.55	19.45	24.59	19.5	-5.09	34.06
	F2#163	24.64	24.69	20.54	20.55	24.67	20.55	-4.12	17.39
	F2#187	25.28	25.48	19.61	19.56	25.38	19.59	-5.80	55.52
	F2#191	24.29	24.33	18.33	18.29	24.31	18.31	-6.00	64.00
	F2#222	25.6	25.53	20.39	20.51	25.57	20.45	-5.12	34.66
	F2#244	24.5	24.62	18.95	18.99	24.56	18.97	-5.59	48.17
	F2#246	24.74	24.81	19.31	19.52	24.78	19.42	-5.36	41.07
	F2#247	25.05	25.25	19.59	19.61	25.15	19.6	-5.55	46.85
	F2#255	25.53	25.82	20.24	20.31	25.68	20.28	-5.40	42.22
	F2#264	25.69	25.64	19.25	19.29	25.67	19.27	-6.40	84.16
**F2-Lov-1 type**	F2#6	24.88	24.9	18.98	18.98	24.89	18.98	-5.91	60.13
	F2#9	24.97	24.95	19.09	19.08	24.96	19.09	-5.88	58.69
	F2#15	25.5	25.43	18.91	18.9	25.47	18.91	-6.56	94.35
	F2#20	25.9	25.71	20.11	20.09	25.81	20.1	-5.71	52.16
	F2#27	25.26	24.97	21.01	20.95	25.12	20.98	-4.14	17.57
	F2#66	25.48	25.03	18.97	19	25.26	18.99	-6.27	77.17
	F2#67	25.25	25.15	19.1	19.07	25.2	19.09	-6.12	69.31
	F2#76	26.22	26.21	20.69	20.56	26.22	20.63	-5.59	48.17
	F2#80	24.98	24.87	20.7	20.48	24.93	20.59	-4.34	20.18
	F2#101	25.17	27.64	19.01	19.04	26.41	19.03	-7.38	166.57
	F2#108	24.69	24.5	19.22	19.13	24.6	19.18	-5.42	42.81
	F2#109	26.18	26.15	18.9	18.92	26.17	18.91	-7.26	152.75
	F2#110	24.88	24.69	19.01	19.01	24.79	19.01	-5.78	54.76
	F2#164	24.71	24.8	19.54	19.64	24.76	19.59	-5.17	35.88
	F2#218	25.77	25.85	19.7	19.67	25.81	19.69	-6.13	69.79
	F2#220	25.11	25.02	18.53	18.55	25.07	18.54	-6.53	92.09
	F2#235	25.43	25.19	19.71	19.75	25.31	19.73	-5.58	47.84
	F2#251	25.55	25.49	19.33	19.21	25.52	19.27	-6.25	76.11
	F2#252	26.61	26.5	20.67	20.63	26.56	20.65	-5.91	59.92

Supplementary Table 3. Raw data for *FLC* expression in SSF-N414D transgenic plants.

Lines	*UBC*			*FLC*			Average cp		Δcp	*FLC* expression
	cp	cp	cp	cp	cp	cp				
	20.58	20.8	20.7	24.31	24.5	24.3	20.70	24.39	3.69	0.08
	20.53	20.52	20.52	24.87	24.8	24.9	20.52	24.84	4.32	0.05
	20.59	20.7	20.6	24.54	24.5	24.5	20.63	24.51	3.88	0.07
	20.75	20.66	20.7	24.77	24.9	24.8	20.70	24.82	4.12	0.06
Col-SSF/ssf-2	20.56	20.57	20.54	24.28	24.4	24.5	20.56	24.40	3.85	0.07
	20.69	20.86	20.72	24.78	24.8	24.8	20.76	24.79	4.03	0.06
	20.91	20.88	20.81	24.82	25	25	20.87	24.94	4.08	0.06
	21.17	20.98	20.97	24.93	25	25	21.04	24.97	3.93	0.07
	21.06	21.19	21.03	25.18	25.2	25.2	21.09	25.18	4.08	0.06
	20.72	20.76	20.75	23.43	23.5	23.3	20.74	23.40	2.66	0.16
	21.55	21.42	21.67	25.16	25.2	25.2	21.55	25.20	3.65	0.08
	20.71	20.82	20.77	24.04	24.1	24.1	20.77	24.08	3.31	0.10
	20.64	20.68	20.6	24.07	24.1	24.1	20.64	24.08	3.44	0.09
	20.32	20.5	20.27	23.75	23.9	23.8	20.36	23.82	3.45	0.09
Col-SSF-N414D/ssf-2	20.66	21.07	20.6	24.46	24.6	24.5	20.78	24.50	3.72	0.08
	20.85	20.9	20.85	24.63	24.7	24.7	20.87	24.67	3.80	0.07
	20.62	20.56	20.59	24.44	24.3	24.3	20.59	24.35	3.76	0.07
	20.97	21.07	21.05	24.81	24.7	24.8	21.03	24.78	3.75	0.07
	20.74	20.82	20.78	24.31	24.3	24.3	20.78	24.31	3.53	0.09
	21.05	21.19	21.16	24.67	24.6	24.6	21.13	24.62	3.49	0.09
	20.78	20.93	20.88	24.58	24.6	24.6	20.86	24.59	3.72	0.08

Supplementary Table 4. List of SSF-GFP mass spectrometric data.

**Gene name**	**GFP**	**SSF**	**CUL1**	**CAND1**
Gene ID	n/a	At2g47310	At4g02570	At2g02560
Col-1	0	0	0	0
Col-2	0	0	0	0
Col-3	0	0	0	0
414N-GFP-1	598	973	10	19
414N-GFP-2	506	785	6	9
414N-GFP-3	547	845	4	13
414D-GFP-1	396	602	3	5
414D-GFP-2	381	590	3	4
414D-GFP-3	385	610	3	5
