# Supplementary material for: Molecular variation in a functionally divergent homolog of FCA regulates flowering time in Arabidopsis thaliana
Source: Nat Commun. 2020 Nov 17;11:5830. doi: 10.1038/s41467-020-19666-0 (PMC7673134; doi:10.1038/s41467-020-19666-0)
Supplement: Supplementary file 4 — Description of Additional Supplementary Files [file 41467_2020_19666_MOESM4_ESM.pdf]

Description of Additional Supplementary Files

File name: Supplementary Data 1
Description: List of the accessions used for GWAS in this study

File name Supplementary Data 2
Description: List of the primers used in this study

File name Supplementary Data 3
Description: Deduced protein sequences of SSF homologs from species used for the phylogenetic analysis.

File name Supplementary Data 4
Description: Alignment of SSF and FCA-like proteins from different plant species.
